# How Geometry Affects Sensitivity of a Differential Transformer for Contactless Characterization of Liquids

**DOI:** 10.3390/s21072365

**Published:** 2021-03-29

**Authors:** Marc Berger, Anne Zygmanowski, Stefan Zimmermann

**Affiliations:** Department of Sensors and Measurement Technology, Institute of Electrical Engineering and Measurement Technology, Leibniz University Hannover, 30167 Hannover, Germany; zygmanowski@geml.uni-hannover.de (A.Z.); zimmermann@geml.uni-hannover.de (S.Z.)

**Keywords:** differential transformer, contactless measurement, filed penetration depth, magnetic coupling, PCB coil, magnetic induced conductivity measurement

## Abstract

The electrical and dielectric properties of liquids can be used for sensing. Specific applications, e.g., the continuous in-line monitoring of blood conductivity as a measure of the sodium concentration during dialysis treatment, require contactless measuring methods to avoid any contamination of the medium. The differential transformer is one promising approach for such applications, since its principle is based on a contactless, magnetically induced conductivity measurement. The objective of this work is to investigate the impact of the geometric parameters of the sample or medium under test on the sensitivity and the noise of the differential transformer to derive design rules for an optimized setup. By fundamental investigations, an equation for the field penetration depth of a differential transformer is derived. Furthermore, it is found that increasing height and radius of the medium is accompanied by an enhancement in sensitivity and precision.

## 1. Introduction

In certain applications, it is of great importance to determine the electrical and dielectric properties of a sample contactless in order to avoid sample contamination by the sensing system. Examples of these applications are the contactless blood conductivity measurement in medical technology as a measure of the sodium concentration or the contactless determination of the polarizability of a medium as a measure of the biomass in biotechnology. Since the sample or medium is often in materials with low permittivity such as polymers or glasses in these applications, contactless capacitive sensors usually suffer from poor penetration of the electric field through these materials. Thus, the sample is only exposed to a small amount of electric field, resulting in a lower sensitivity. For example, in [1], an investigation was made to determine the biomass through a polymer foil of a single-use bioreactor by a coplanar stripe line. Since most of the electric field was concentrated in the foil, these results were not promising. However, there are systems like capacitively coupled contactless conductivity detection (C^4^D) [2], allowing for capacitive measure through a usually special fused-silica capillary with a small cross-section [3,4]. Nevertheless, due to the small cross-section of the capillary, C^4^D is not suitable for in-line measurements where higher fluxes of the medium is expected. In addition, due to the above-mentioned poor penetration properties of the electric field through the capillary, the capillary wall can have a dominant influence on the impedance in this principle depending on the conductivity of the sample solution [5].

Inductive conductivity measuring systems can penetrate the materials much better, causing the sample to be exposed to a relatively high magnetic field strength. A typical configuration of inductive conductivity sensors consists of two coils immersed in and coupled by the liquid under test [6]. Due to the immersion of this sensor, it is not a contactless determination and is therefore not suitable for applications requiring the prevention of sample contamination. Single-coil configurations can be used for contactless measurement. Here, the coil is placed close to the sample and the coil inductance is used as the measure, changing with varying electrical and dielectric properties of the sample [7]. However, the measurement signal is dominated by the primary field, resulting in an output voltage with a strong offset. Small changes in the secondary field *B*_S_ due to weak induced currents inside the medium are thus challenging to detect [1].

In this work, we use a differential setup consisting of three coils, giving a differential transformer. Differential transformers are already used in various sensing applications. One prominent example is the linear variable differential transformer, usually consisting of a movable magnetic soft iron or ferrite core inside a configuration of three coaxial coils [8,9,10,11]. These coils are arranged symmetrically to each other. The middle coil is the primary coil, excited by an alternating voltage of constant amplitude. The two outer coils are the secondary coils, which are connected differentially to each other. Due to this arrangement, a relative displacement of the ferrite core to the coils results in a disturbance of the symmetry, inducing a measurable output voltage at the differentially connected secondary coils. The output voltage corresponds very well with the displacement of the ferrite core, giving a high resolution and low noise displacement, position, or force sensors [12]. This type of application is well known and often used in practice [13,14,15,16,17].

**Figure 1:**
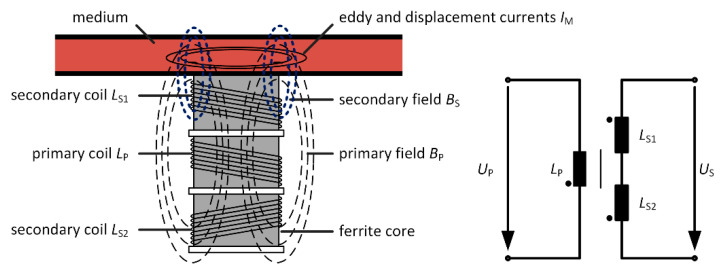
Depiction of the differential transformer consisting of three fixed coils on a ferrite core for the analysis of the electrical and dielectric properties of a medium in a measuring chamber or tubing (loaded) and its electrical equivalent circuit of the unloaded transformer. The secondary coils *L*_S1_ and *L*_S2_ are connected differentially in series.

In another setup, the ferrite core is fixed. If now a sample or medium is placed closer to one of the two secondary coils of the differential transformer, the asymmetry allows for measuring the electrical properties, such as the conductivity *κ*, and the dielectric properties, such as the polarizability *ε*′, of the medium. Figure 1 schematically shows such a differential transformer and the equivalent electric circuit of the connected coils of an unloaded differential transformer. The measurement effect is based on eddy and displacement currents *I*_M_ within the medium, induced by the alternating primary field *B*_P_. *B*_P_ is generated by the primary coil *L*_P_, excited with *U*_P_. These induced currents generate a secondary field *B*_S_, counter-directed to the primary magnetic field. Due to the asymmetrical arrangement of the medium to the differential transformer, the secondary field better couples with the secondary coil *L*_S1_ closer to the sample than with *L*_S2_ and therefore induces a larger voltage into *L*_S1_. As a result, an output voltage *U*_S_ can be measured at the output of the differentially connected secondary coils. Because of the differential setup of *L*_S1_ and *L*_S2_ and the symmetrical design of the two secondary coils relative to the primary coil *L*_P_, the primary magnetic field does not induce a measurable voltage at the output of the connected secondary coils. This is a great advantage compared to configurations where only one single coil is used as described above; enabling even very low eddy and displacement currents *I*_M_ inside the medium to be detected. As pointed out earlier, magnetic fields can easily penetrate plastics and glass. Therefore, this measuring method is well suited for the contactless determination of the electrical and dielectric properties of liquids located inside a plastic or glass measuring (flow-through) chamber. Therefore, this measurement method has potential in biotechnology and medical technology for the contactless determination of biomass within single-use bioreactors [1,18] or for obtaining tissue information [19,20,21,22,23]. In addition, the differential transformer approach is investigated with respect to continuous in-line monitoring of the sodium concentration in human blood, by measuring the blood plasma conductivity, mainly influenced by the sodium concentration [24,25,26,27]. Continuous monitoring of plasma sodium concentration is particularly important in continuous renal replacement therapy, especially in patients with severe dysnatremia, since both a large deviation from the physiological plasma sodium level [26,28,29,30] and a rapid change in the concentration can lead to dangerous complications such as central pontine myelinolysis [31,32]. Continuous monitoring of this parameter enables the clinician to intervene as early as possible by individualized dialysis therapy [33,34,35]. The differential transformer is well suited for this application because it is based on a contactless measurement principle and thus avoids any risk of blood contamination during in-line measurement. In preclinical studies with reconfigured human blood in a dialysis system, we could already show in cooperation with the University Medical Center Göttingen, that the differential transformer can be used to monitor the sodium concentration over a long period of time [36,37].

In [1] it was demonstrated that the output voltage *U*_S_ of the differential transformer can be divided into a real part, depending on the polarizability *ε*′ of the medium, and an imaginary part indicated by the imaginary unit *j*, depending on the conductivity *κ* and the dielectric losses *ε*″, as shown in Equation (1). This division of the output voltage is another advantage offered by the differential three-coil configuration.

(1) $$U_{S}=U_{P}\left(-\omega^{2}K\varepsilon^{\prime }+j\;\omega K\left(\kappa -\omega \varepsilon^{\prime \prime }\right)\right)$$

*ω* is the angular frequency. *U*_P_ is the supplied input voltage of the primary coil *L*_P_. *K* describes the magnetic coupling between *L*_P_ and the medium as well as the medium and the secondary coils *L*_S1_ and *L*_S2_. Solutions to describe the magnetic coupling between wires and coils can be found, i.e., in [38]. In [39], an exact solution of the mutual inductance of two coils is given. However, for the case of the differential transformer, we have presented a mathematical model describing the coupling of the coils involving the medium in [37]. This mathematical model will be discussed in more detail in Section 3.1.2.

Due to the growing number of possible applications of a differential transformer for the characterization of liquids and thus increasing interest for this sensing concept, it is further investigated here. The objective of this work is to determine the basic effect of the sample geometry, such as the radius *r*_M_ of the medium and the height *h*_M_ of the medium, on the output signal of the differential transformer. Besides gaining a basic understanding, e.g., the effective sample volume, this knowledge shall additionally support the optimization of the design of new measurement chambers to achieve a maximum sensitivity of the differential transformer and thus improve the *SNR*.

## 2. Materials and Methods

### 2.1. PCB-Differential Transformer

**Figure 2:**
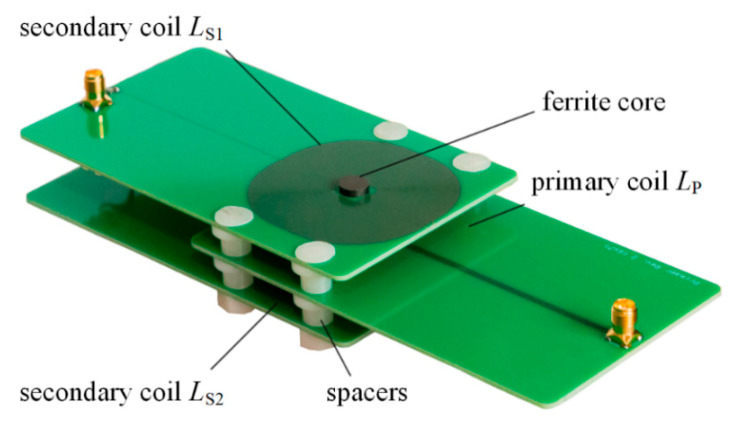
Photograph of the used differential transformer made of three printed circuit board (PCB) coils. The differential connection of *L*_S1_ and *L*_S2_ is realized via two wires (not seen).

**Figure 3:**
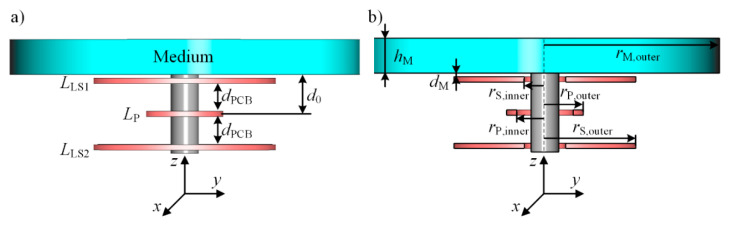
(**a**) Illustration of the CST-EM Studio simulation model. The number of windings of the primary coil excited with 1 V_PP_ is 42. The secondary coils *L*_S1_ and *L*_S2_ have 542 windings, and the direction of the winding of *L*_S1_ is opposite to *L*_S2_. The distance *d*_PCB_ between the coils is 8 mm and the height of each PCB coil is 1.5 mm. *d*_0_ is the distance between the medium and the primary coil. The coordinate system is defined as having the *z*-axis longitudinal to the ferrite core and the *x*- and *y*-axes in the radial direction. (**b**) Cross-section of the differential transformer with the height *h*_M_ and the outer radius *r*_M,outer_ of the medium or the sample, respectively. *r*_P,inner_ and *r*_P,outer_ are the inner and outer radii of the primary coil *L*_P_. Accordingly, *r*_S,inner_ and *r*_S,outer_ are the inner and outer radii of the secondary coils *L*_S1_ and *L*_S2_.

For the experimental characterization, we use a differential transformer consisting of three planar printed circuit boards (PCB) containing conductive tracks of a height of 35 μm forming coils on a ferrite core. A photography of the used differential transformer is shown in Figure 2. All geometric dimensions are illustrated in Figure 3.

The planar PCB coils enable the distance *d*_PCB_ between the primary coil *L*_P_ and the secondary coil *L*_S1_ as well as between the primary coil *L*_P_ and the secondary coil *L*_S2_ to be set precisely. By positioning these planar PCB coils at a defined distance from each other, the magnetic coupling between the coils and the medium can be affected. This can have positive effects on the sensitivity, as already discussed in [37]. Here, *d*_PCB_ is set to 8 mm via spacers. In addition, when the medium is positioned above *L*_S1_, the flat design of these planar PCB coils enables all windings of the secondary coil *L*_S1_ to be close to the medium and hence *L*_S1_ is penetrated strongly by secondary field *B*_S_. This results in a higher sensitivity of the differential transformer. All PCB coils were realized on a six-layer board, having the dimensions 90 × 120 × 1.5 mm. The ferrite core has a radius of 4 mm and a relative permeability *μ*_r_ of 300. The length of the ferrite core is 22 mm, penetrating all three PCB coils, as the total height of the three stacked coils is 20.5 mm. The upper and lower coils are the secondary coils *L*_S1_ and *L*_S2_ with a total number of windings *n*_S_ of 542 each, 91 windings on the top and bottom layer and 90 windings on the four middle layers. The track width of *L*_S1_ and *L*_S2_ is 0.1 mm with a clearance of 0.125 mm as well as an inner coil radius *r*_S,inner_ of 6 mm resulting in an outer coil radius *r*_S,outer_ of 26 mm. The inductivities *L*_S1_ and *L*_S2_ are each 23.8 mH with a DC resistance of 320 Ω. *L*_S1_ and *L*_S2_ are connected in series differentially via two wires. The middle coil is the primary coil *L*_P_ with a total of *n*_P_ = 42 windings, giving seven windings per layer. The track width is 0.3 mm with a clearance of 0.125 mm. The outer coil radius *r*_P,outer_ is 11 mm and the inner coil radius *r*_P,inner_ is 8.1 mm. However, the geometrical dimensions of the primary and secondary coils will be discussed in detail later in this work. The inductance of *L*_P_ is 31.9 μH with a DC resistance of 3.4 Ω. The primary coil and the upper secondary coil *L*_S1_ have SMA connectors for the electrical connection. As can be seen from Equation (1), the output voltage *U*_S_, and thus the sensitivity of the differential transformer, depends linearly on the voltage *U*_P_ applied to the primary coil *L*_P_. In the investigations presented here, we excite *L*_P_ with 1 V_PP_ peak to peak, meaning the following sensitivity data always refer to this supply voltage. The choice of 1 V_PP_ has the advantage of enabling the sensitivities for other voltage *U*_P_ to be calculated easily and quickly, and making the obtained sensitivities comparable with previous publications [36,37]. In addition, Equation (1) reveals a dependency of the output voltage *U*_S_ and thus the sensitivity to the angular frequency *ω* or frequency *f*, respectively. It can be seen that a high frequency is generally desirable, as this increases the sensitivity. However, due to the always present stray capacitance between the windings of the coil, the frequency cannot be increased arbitrarily. These stray capacitances cause a resonant frequency. Above this resonance frequency, the coil has no inductive behavior. Therefore, *f* must always be lower than the resonance frequency. In [40], a possibility is described to estimate these stray capacitances and thus the resonance frequency a priori. Since we have coils with a rectangular cross section of the conductor and each coil has six layers, but in [40] only coils with isolated round conductors and at most three layers are considered, we have determined the resonant frequency experimentally. For the secondary coils, this is 250 kHz. Thus, at *f* = 155 kHz, the secondary coils behave as inductances. In all experiments, the sample is placed in different compartments defining the sample geometry on top of *L*_S1_.

### 2.2. Test Solution

Deionized water (DI water) with different concentrations of sodium chloride (NaCl) is used for the experimental characterization of the differential transformer. The concentration is varied between 100 mmol/L and 150 mmol/L, as this range covers the clinically relevant pathological concentration range of sodium in blood serum, which is relevant for the above-mentioned monitoring during continuous renal replacement therapy [26,29,31,32,33,34]. Although the concentration generally has a non-linear impact on the sample conductivity [41], it could be shown in [36] that the imaginary part of the output voltage *U*_S_ of the differential transformer depends linearly on the concentration within this narrow range. The sensitivity *S*_c_ is determined by using the imaginary part of the output voltage *Im*{*U*_S_} at two different concentrations (e.g., *c*_1_ = 100 mmol/L and *c*_2_ = 150 mmol/L) according to Equation (2).

(2) $$S_{c}=\frac{dIm\left\{U_{S}\left(c\right)\right\}}{dc}=\frac{Im\left\{U_{S}\left(c_{2}\right)-U_{S}\left(c_{1}\right)\right\}}{c_{2}-c_{1}}$$

### 2.3. Simulation Model

In addition to the experimental tests, the setup is simulated using the CST-EM Studio. The basic simulation model is shown in Figure 3a, representing the differential transformer described above. *d*_0_ is the distance between the primary coil *L*_P_ and the bottom of the medium and is 11.25 mm.

The coils are created in CST-EM Studio from a rectangular cross-section rotated around the *z*-axis. Using the coil generation tool, a number of windings, a wire resistance as well as a winding direction can be assigned to this coil. However, further geometrical factors, such as the conductor cross-section and filling factor, are not taken into account, whereby the winding-to-winding stray capacitances are also neglected. Therefore, it should be noted that this model is only valid at frequencies below the resonant frequency mentioned above and the measurements can only be compared with the simulations in this case. The direction of winding of *L*_S1_ is inverted to the direction of winding of *L*_S2_. Thus, the sum of the induced voltages into *L*_S1_ and *L*_S2_ gives the output voltage *U*_S_ of the differential transformer. The voltage induced into each secondary coil can be derived from CST-EM Studios and divided into real and imaginary part. In contrast to the experimental tests, the conductivity *κ* of the medium can be changed directly in the simulation model. The primary coil *L*_P_ is excited with 1 V_pp_ at a frequency of 155 kHz. The secondary coils *L*_S1_ and *L*_S2_ are excited with a current of 0 A, corresponding to the condition of an ideal voltage measurement. *Im*{*U_S_*} depends linearly on *κ*, as expected from Equation (1) [37]. The conductivity of the medium is varied between 1 S/m and 2 S/m, since this corresponds approximately to the conductivity of the blood [42]. This allows the sensitivity *S*_κ_ of the differential transformer to be determined in terms of the conductivity *κ* as input variable according to Equation (3).

(3) $$S_{\kappa }=\frac{dIm\left\{U_{S}\left(\kappa \right)\right\}}{d\kappa }=\frac{Im\left\{U_{S}\left(\kappa_{2}\right)-U_{S}\left(\kappa_{1}\right)\right\}}{\kappa_{2}-\kappa_{1}}$$

For all following sections, the coordinate system is defined as shown in Figure 3. The *z*-axis points in the longitudinal direction, i.e., along the ferrite core. The *x*- and *y*-axis point in radial direction.

Figure 3b shows a cross-section of the differential transformer in the *y*-*z*-plane. Depending on the parameter of interest, here, the height *h*_M_ in *z*-direction or the outer radius *r*_M,outer_ of the medium in *x*-*y*-direction are varied. In Section 3.1.2, the radius *r*_S,outer_ of *L*_S1_ and *L*_S2_ are also changed.

## 3. Results and Discussion

### 3.1. Impact of the Sample Geometry on the Sensitivity

An important issue that was not considered in previous investigations [18,37] and thus is not included in e.g., Equation (1), is the relationship between the sample geometry and the sensitivities *S*_κ_ and *S*_c_ and consequently the signal-to-noise ratio *SNR* of the differential transformer. Although other factors, such as the diameter or permeability of the ferrite core, also affect the sensitivity, in this work we will focus on rotationally symmetric samples as well as on the radius of the primary and secondary coils. Especially, the radius *r*_M_ and the height *h*_M_ and therefore the volume of the sample is of particular interest, since often, the sample volume is defined by certain specifications coming from the application. For example, such restrictions can be found in the continuous in-line monitoring of the sodium concentration of the blood in extracorporeal circuits during dialysis treatment, where, in addition to flow requirements, the sample volume should be kept as low as possible in order to draw as less blood as possible from the patient. Therefore, the effect of the height *h*_M_ and the outer radius *r*_M,outer_ of a cylindrically shaped sample compartment on the sensitivities *S*_κ_ and *S*_κ_ and the *SNR* should be investigated. Thus, the objective is to determine to what degree an increase of these parameters can improve *S*_κ_, *S*_c_, and *SNR* while considering the sample volume limitations in order to derive design rules for the sample compartment.

**Figure 4:**
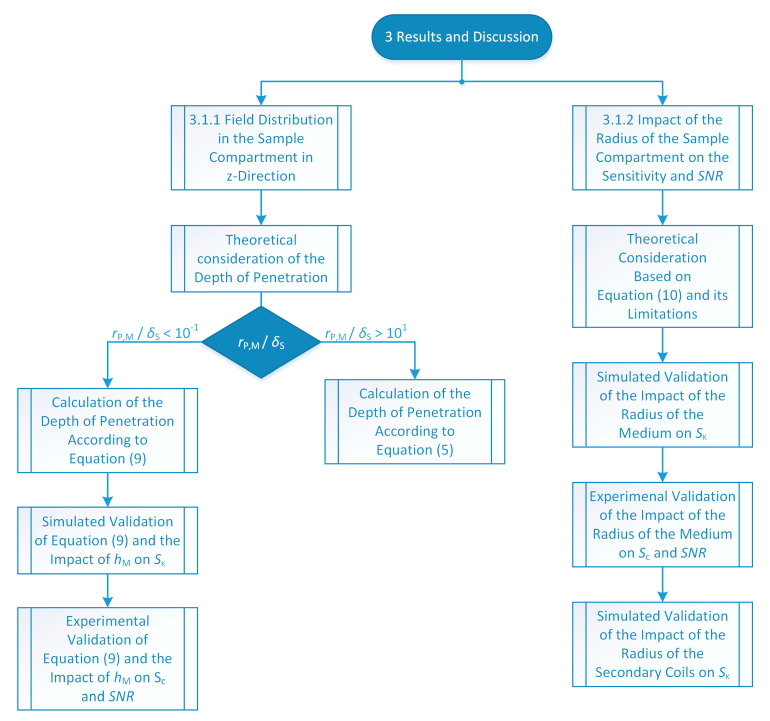
Flowchart of the investigations conducted in Section 3.

Therefore, as illustrated in Figure 4, Section 3.1.1 deals with the investigation of the field distribution in the sample compartment in *z*-direction and Section 3.1.2 studies the impact of the radius of the sample compartment on the sensitivity and the signal to noise ratio *SNR*. First, in Section 3.1.1 a theoretical consideration of the depth of penetration of the field into the medium is given. Here, it is found that a case distinction must be made. If the ratio of the mean radius of the primary coil *r*_P,M_ to the skin depth or standard depth of penetration *δ*_S_ is greater than 10, the equation for *δ*_S_ can be used to calculate the depth of penetration. This case is well known and already described in detail in the literature. Thus, no further consideration is made for this case. However, for most technically relevant liquids, the ratio *r*_P,M_/*δ*_S_ is less than 10^−1^. For this case, we present a new equation enabling the true depth of penetration to be determined. Then, this new equation is verified with the simulation model. In addition, the influence of the height of the medium *h*_M_ on the sensitivity *S*_κ_ is investigated. Subsequently, these results are validated experimentally and the influence of *h*_M_ on the signal-to-noise ratio *SNR* is investigated. In Section 3.1.2, first, the influence of the radius of the medium on the sensitivity is investigated by using simplified theoretic considerations. The limits of this simplified theory are shown. Afterwards, for a more detailed consideration, the influence of the radius of the medium on the sensitivity is analyzed with the simulation model. These results are subsequently validated experimentally by measurements using the constructed differential transformer. In addition, the influence on the *SNR* is also investigated here. Since these findings show that the radius of the secondary coils affects the sensitivity curve, the simulation model validated by the experimental investigations is then used to evaluate this influence in detail. All the data shown in the following section are included in it. Further information on data availability can be found in the Supplementary Materials statement. The data presented in this study are available on request from the corresponding author.

#### 3.1.1. Field Distribution in the Sample Compartment

The magnitude of the electromagnetic field generated by the excited primary coil *L*_P_ decreases in *z*-direction. As the density of the eddy and displacement currents *I*_M_, measured by the secondary coils *L*_S1_ and *L*_S2_, are directly related to the electromagnetic field inside the medium according to Maxwell’s law [43], the current density within the medium also decreases along the *z*-direction. Thus, above a certain height *h*_M_ of the sample compartment, the induced current density is too low for a measurable contribution to the secondary magnetic field and hence to the output voltage *U*_S_. Therefore, a further increase of *h*_M_ does not improve sensitivity. The penetration depth of electromagnetic field and thus the eddy currents in the sample is typically described by solving the field diffusion equation of a planar electromagnetic wave, propagating e.g., in *z*-direction and reaching a conducting half space at *z* = 0. Solving this diffusion equation results in Equation (4) [43,44,45], where this is valid for *z* ≥ 0. For *z* < 0, *J*(*z*) is zero.

(4) $$J\left(z\right)=J\left(0\right)\cdot e^{-\frac{z}{\delta_{S}}}$$

Here, *J*(*z*) is the current density depending on the distance in *z*-direction. *J*(0) is the initial value of the current density for *z* = 0. By replacing *J* with the magnetic field *B* or electrical field *E*, Equation (4) also describes the decrease of this parameter inside a medium. For eddy current sensors, the parameter *δ*_S_ is typically called standard depth of penetration or skin depth in the scientific literature [46]. At *z* = *δ*_S_ the current density has attenuated to 1/*e* or about 37% respectively of the initial value at *z* = 0. The standard depth of penetration *δ*_S_ only depends on material parameters such as the conductivity *κ* and the permeability *μ* as well as frequency *f* of the field and can be calculated according to Equation (5) [43].

(5) $$\delta_{S}=\sqrt{\frac{1}{\pi f\mu \kappa }}$$

For typical applications of the differential transformer, the conductivity measured in Siemens per meter is in the single-digit range. For example, the conductivity of blood for monitoring the sodium concentration is between 1 S/m and 2 S/m [42]. In the case of water quality monitoring, the conductivity can be slightly higher, e.g., about 5 S/m for seawater [47]. The magnetic properties *μ* of a sample is the product of the permeability of free space *μ*_0_ and the relative permeability *μ_r_* of the corresponding medium, where usually for the application presented here the relative permeability can be considered as *μ_r_* = 1, so that *μ* = *μ*_0_ applies. For a differential transformer driven at a frequency *f* of 155 kHz, Equation (5) would yield a standard depth of penetration *δ*_S_ of about 0.9 m to 1.3 m for conductivities between 1 S/m and 2 S/m, respectively. However, these depths of penetration are not to be expected in practical applications.

In order to calculate the true depth of penetration *δ*_T_, Dodd et al. has described the depth of penetration of an eddy current sensor for non-destructive material testing using an analytical solution of the vector potential *A* [48]. The excited coil is an axisymmetric circular coil with a rectangular cross-section in the plane of the symmetry axis and radial axis. The mean coil radius *r*_P,M_ is the arithmetic mean value between the outer and inner radius of the coil. Since these sensors have similarities to the differential transformer with respect to the depth of penetration, the findings can be transferred. The very complex description using the vector potential *A* was analyzed by Mottl [49]. It was found that a calculation of the standard depth of penetration *δ*_S_ according to Equation (5) is valid as long as the ratio between the mean coil radius *r*_P,M_ of the excited coil and the calculated standard depth of penetration *δ*_S_ is greater than 10 (*r*_P,M_/*δ*_S_ > 10). For a smaller ratio, the actual depth of penetration deviates from that in Equation (5). The reason is the assumption of planar waves to derive Equation (4). A planar wave implies, among other things, that the electromagnetic field has a constant amplitude along the propagation direction and is not attenuated without the presence of a medium. The attenuation of planar waves is solely due to the properties of the medium. To obtain an understanding why the true depth of penetration *δ*_T_ can be significantly lower and to have a much easier approach to estimate the *δ*_T_ than in [48], we use the Biot–Savart law. This allows to determine the magnetic field *B*(*z*) of a coil that is rotationally symmetric to the *z*-axis, along the *z*-direction. Since the Biot–Savart law neglects the spatial expansion of the coil, it is assumed for a coil with a rectangular cross-section that all *n*_P_ windings are concentrated at the mean radius *r*_P,M_. In this case, the *B*(*z*)-field along the symmetry axis (*z*-axis) of a coil excited with the current *I*_P_ can be described by Equation (6).

(6) BP(z)=μ2rP,M2 IP nP(rP,M2+z2)32

Equation (6) shows that the *B*-field decreases with increasing distance *z* from coil origin (*z* = 0). The decrease depends on the mean coil radius *r*_P,M_. Calculating the *B*-field of a coil in free space and without the presence of any sample according to Equation (6) at the position *z* = *δ*_S_ and normalizing the *B*-field to the initial value of the *B*-field at the position *z* = 0, yields Equation (7).

(7) $$\frac{B_{P}\left(\delta_{S}\right)}{B_{P}\left(0\right)}={\left(\frac{1}{1+{\left(\frac{\delta_{S}}{r_{P,M}}\right)}^{2}}\right)}^{\frac{3}{2}}$$

**Figure 5:**
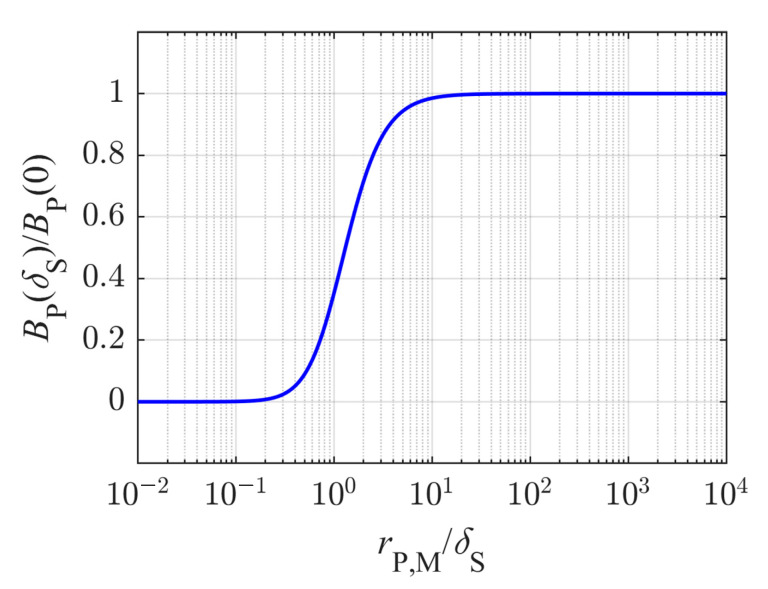
Ratio of the magnetic field strength *B*_P_ of the primary coil at the point z = *δ*_S_ to the initial field strength at the point *z* = 0 versus the ratio of the mean primary coil radius *r*_P,M_ to *δ*_S_. *δ*_S_ is the standard depth of penetration calculated according to Equation (5) to determine the skin depth of planar waves within a medium.

By plotting Equation (7) versus the ratio *r*_P,M_/*δ*_S_, Figure 5 is obtained, where *r*_P,M_/*δ*_S_ can be varied by changing the primary coil radius *r*_P,M_ and the standard depth of penetration *δ*_S_ depending on the sample conductivity and the excitation frequency according to Equation (5).

For values of *r*_P,M_/*δ*_S_ ≥ 10, it can be seen that the ratio *B*_P_(*δ*_S_)/*B*_P_(0) tends towards the value 1, meaning that the *B*-field in free space can be seen almost constant up to *z* = *δ*_S_, meeting the conditions to be considered as a planar wave and supporting the results obtained from [49]. Hence, the attenuation within the medium can be described by Equations (4) and (5), as the medium is the dominant reason for lowering the current density or field strengths. To reach the standard depths of penetration *δ*_S_ of about 1 m for the above-calculated sodium monitoring in blood would require a technically unreasonable coil radius of about 10 m. The mean coil radius *r*_P,M_ of the differential transformer used here is about 9.55 mm, giving a ratio *r*_P,M_/*δ*_S_ of about 10^−2^. At this point, Equation (7) approaches zero, indicating that the *B*-field of the coil has nearly decreased to zero at *z* = *δ*_S_ (with *δ*_S_ ≈ 1 m) only due to the coil geometry. Thus, it can be assumed that the decrease of the field is dominated by the coil geometry of the excited coil while the attenuation due to the electromagnetic properties of the medium has a negligible impact on the field distribution. In order to estimate the true depth of penetration *δ*_T_ in the case that the decrease of the *B*-field is dominated by the coil geometry, we present a very simple approach using the Biot–Savart law. *δ*_T_ is defined similar to δ_S_ as the depth of penetration, at which the *B*-field and thus also the current density *J* has declined to the value 1/*e* i.e., 37% relative to the initial value at *z* = 0. Therefore, the ratio *B*_P_(*δ*_T_ + *d*_0_)/*B*_P_(*d*_0_) is determined by using Equation (6) and is set equal to 1/*e*. *d*_0_ is the distance in *z*-direction between the primary coil and the medium, see Figure 3, and is about 11.25 mm for the differential transformer used here. As a result, Equation (8) is obtained.

(8) $$\frac{B_{P}\left(\delta_{T}+d_{0}\right)}{B_{P}\left(d_{0}\right)}=\frac{1}{e}=\frac{\;\mu \cdot r_{P,M}^{2}\cdot I_{P}}{2{\left(r_{P,M}^{2}+{\left(\delta_{T}+d_{0}\right)}^{2}\right)}^{\frac{3}{2}}}\cdot \frac{2{\left(r_{P,M}^{2}+d_{0}^{2}\right)}^{\frac{3}{2}}}{\mu \cdot r_{P,M}^{2}\cdot I_{P}}\;={\left(\frac{r_{P,M}^{2}+d_{0}^{2}}{r_{P,M}^{2}+{\left(\delta_{T}+d_{0}\right)}^{2}}\right)}^{\frac{3}{2}}$$

Solving Equation (8) for the true depth of penetration *δ*_T_ leads to Equation (9), enabling the depth of penetration of eddy current sensors to be estimated in a simple way. This equation is valid for *r*_P,M_/*δ*_S_ < 10^−1^, since here the decrease of the field strength of the *B*-field is mainly caused by the coil geometry and the attenuation due to the medium can be neglected.

(9) $$\delta_{T}=\sqrt{\left(\left(r_{P,M}^{2}+d_{0}^{2}\right)\cdot e^{\frac{2}{3}}-r_{P,M}^{2}\right)}-d_{0}$$

Calculating the true depth of penetration *δ*_T_ for the differential transformer used here with *r*_P,M_ = 9.55 mm and *d*_0_ = 11.25 mm, Equation (9) results in *δ*_T_ = 7 mm, which is significantly lower than the *δ*_S_ of approximately 1 m due to the relatively low conductivity of the medium *κ* (e.g., blood) and excitation frequency *f* according to Equation (5).

In order to confirm the calculated *δ*_T_, numerical computer simulations are conducted using the CST-EM Studio model described in Section 2.3. Subsequently, these simulations are validated by measurements. Besides the true depth of penetration *δ*_T_ of the eddy currents and the resulting current distribution of the current density *J*_M_ within the medium, the dependence of the sensitivity on the height *h*_M_ of the medium is of particular interest. Since the sensitivity is proportional to the total eddies and displacement currents *I*_M_ induced into the medium, i.e., to the integral of *J*_M_ along the *z*-axis and *J*_M_ decreases with increasing *h*_M_, it can be expected that *S*_κ_ initially rises with increasing height *h*_M_ of the medium and starts to saturate at a certain point. As the eddy current density has decreased to 37% of the initial value at *δ*_T_, the sensitivity *S*_κ_ can only increase by 37% by further increasing *h*_M_, i.e., *S*_κ_ has reached 67% of the maximum value. Considering an exponentially decreasing depth of penetration, the point 3∙*δ*_S_ is called the effective depth of penetration. At this point, the current density is already attenuated by about 95%. Therefore, all currents induced above the effective depth of penetration have only a negligible effect. The definition of the effective depth of penetration is also assumed here for the true depths of penetration determined according to Equation (9), so that 3∙*δ*_T_ applies to the effective depth of penetration. A calculated true depth of penetration *δ*_T_ of 7 mm, results in an effective depth of penetration 3∙*δ*_T_ = 21 mm.

**Figure 6:**
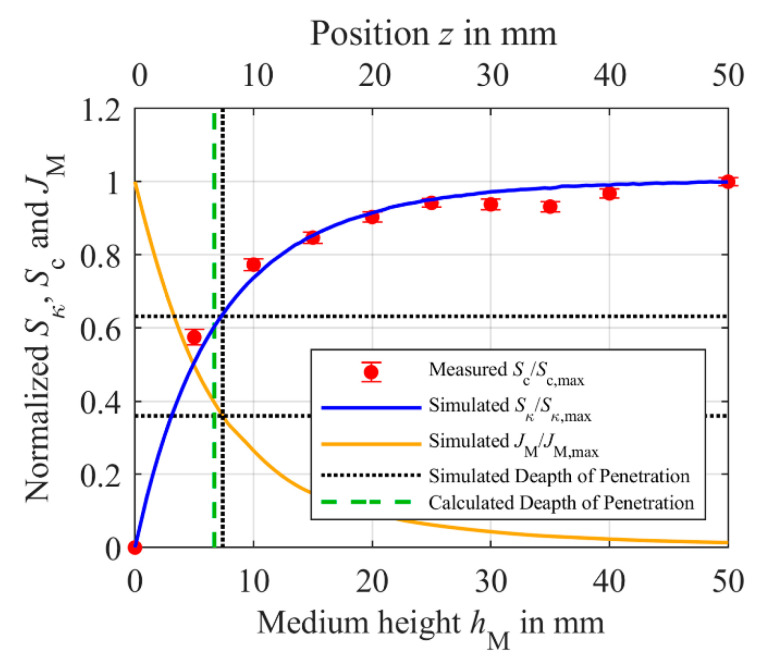
Normalized simulated sensitivity *S*_κ_ (blue solid line) of the differential transformer depending on the height *h*_M_ of the medium (lower *x*-axis), if *h*_M_ is changed from 0 mm to 50 mm (conductivity *κ* of the medium: 1 S/m to 2 S/m). The red dots are the measured sensitivity *S*_c_ with error bar, when *h*_M_ (lower *x*-axis) is varied from 0 mm to 50 mm in 5 mm steps (the sodium concentration is 100 mmol/L and 150 mmol/L). *S*_κ_ is normalized to the maximum sensitivity *S*_κ,max_ = 114.8 μV/S/m. The measured sensitivity *S*_c_ and the error bars are both normalized to *S*_c,max_ = 38.74 mV/mol/L. The orange line represents the current distribution *J*_M_ in *z*-direction (upper *x*-axis) within the sample with *h*_M_ = 50 mm and a conductivity of *κ* = 2 S/m normalized to the maximum *J*_M,max_ of 202.5 mA/m^2^. The true depth of penetration *δ*_T_ obtained from the simulations, at which the sensitivity has reached about 63% or *J*_M_ as decreased to 37% of its maximum value, is 7.4 mm (vertical black dotted line). The true depth of penetration *δ*_T_ calculated using Equation (9) is at 7 mm (green dashed line).

Now, simulations according to the model from Section 2.3 help to verify whether this relationship between the true depths of penetration of the current density is consistent with the dependence of sensitivity on the height *h*_M_ of the medium or sample compartment, respectively. First, the height *h*_M_ of the sample compartment is set to 50 mm, the outer radius *r*_M,outer_ to 47 mm and the inner radius to 0 mm. The conductivity *κ* of the medium is set to 2 S/m and the primary coil is excited with 1 V_PP_ and 155 kHz. Figure 6 shows the resulting curve of the current density within the sample in *z*-direction (orange solid line versus the upper *x*-axis, normalized to the initial *J*_M_ at the bottom of the medium). The intersection of the horizontal black dotted line at 0.37 with the vertical black dotted line gives a simulated true depth of penetration of *δ*_T_ = 7.4 mm. The height *h*_M_ of the medium is now varied from 0 mm to 50 mm, in order to proof that the depth of penetration can also be determined based on the sensitivity of the differential transformer. Each height *h*_M_ is simulated for 1 S/m and 2 S/m, allowing the sensitivity to be determined using Equation (3). The simulated sensitivity *S*_κ_ is shown in Figure 6 (blue solid line versus the lower *x*-axis). The sensitivity is normalized to the maximum *S*_κ,max_ of 114.8 μV/S/m. As can be seen, the sensitivity first rises rapidly with increasing *h*_M_ and then saturates, as expected. 63% of the maximum sensitivity *S*_κ,max_, is reached at the simulated *δ*_T_ of the induced current density *J*_M_ is 7.4 mm. Thus, the true depth of penetration *δ*_T_ of the eddy and displacement currents and the sensitivity *S*_κ_ of the differential transformer are proportional to each other, and *δ*_T_ can therefore be derived from the sensitivity, which is a considerable advantage for determination of *δ*_T_ by measurements, since the sensitivity is much easier to measure than *J*_M_ within the medium. This simulated true depth of penetration *δ*_T_ is close to the calculated *δ*_T_ of 7 mm by Equation (9), which is also shown as the green dashed line in Figure 6.

In order to validate the simulations, the depth of penetration was also measured, using the differential transformer described in Section 2.1. The differential transformer is driven with a peak-to-peak input voltage of 1 V_pp_ and a frequency *f* of 155 kHz. The medium was positioned above the differential transformer within a compartment having a radius of 47 mm. Similar to the simulation; the medium height *h*_M_ was varied from 0 mm to 50 mm. As described in Section 2.2, the sample solution is DI-water containing NaCl with a concentration of *c*_1_ = 100 mmol/L and *c*_2_ = 150 mmol/L, so that the sensitivity *S*_c_ can be calculated according to Equation (2) for each height *h*_M_. The results are shown in Figure 6 as red dots. The measured sensitivity is normalized to the maximum measured *S*_c,max_ of 38.74 mV/mol/L. The measured values are in good agreement with the simulated values validating the simulation and the mathematical model from Equation (9).

Furthermore, an important issue is whether the increased sensitivity can improve the precision and thus the *SNR* of the differential transformer. Therefore, we determined the noise of the output signal by the standard deviation of the imaginary part of *U*_S_, which is averaged 512 times giving a measured value about every 11 s. Table 1 gives the noise measured for different heights *h*_M_ and a concentration of 150 mmol/L NaCl. The standard deviation of the measured concentration can be calculated by the standard deviation of *Im*{*U*_s_} divided by the respective sensitivity *S*_c_. Table 1 reveals that there is no correlation between *h*_M_ and the noise of the imaginary part of *U*_S_. Thus, comparing the standard deviation of *c*_std_ can be reduced from 0.81 mmol/L to 0.44 mmol/L by increasing *h*_M_ due to the increasing sensitivity *S*_c_. The relative error bars normalized to *S*_c,max_ are illustrated in Figure 6.

In summary, the true depth of penetration *δ*_T_ of the differential transformer used here cannot be calculated using Equation (5) due to the small ratio *r*_P,M_/*δ*_S_ of the primary coil radius to the standard depth of penetration. As a result of the relatively small coil radius *r*_P,M_ and the low conductivity of the medium, the magnetic field of the coil and thus the currents induced into the medium have already approached zero before reaching *δ*_S_ due to the coil properties. *δ*_S_ only depends on the frequency *f* of the excited coil and the material properties of the medium, such as the conductivity *κ* and permeability *μ*. Therefore, we introduced Equation (9) estimating the true depth of penetration *δ*_T_ when *r*_P,M_/*δ*_S_ is below 10^−1^. With Equation (9), the true depth of penetration *δ*_T_ of the differential transformer used here, was calculated to 7 mm. The simulation model, validated by measurements, showed a depth of penetration of about 7.4 mm. Hence, Equation (9) is well suited for estimating *δ*_T_. The true depth of penetration *δ*_T_ is particularly important for the design of the measuring chamber, when the sample volume is limited by the application. For *h*_M_ ≤ *δ*_T_, the sensitivity can be significantly improved by increasing *h*_M_. However, by further increasing *h*_M_ the effect of *h*_M_ on *S*_κ_ and *S*_c_ decreases, e.g., for *h*_M_ > 3∙*δ*_T_, increasing *h*_M_ does not noticeably increase the sensitivity. Since the noise of the output signal *Im*{*U*_S_} has shown no inherent correlation with the medium height *h*_M_, an increase in sensitivity by increasing *h*_M_ is associated with an improvement in signal-to-noise ratio *SNR*.

#### 3.1.2. Impact of the Radius of the Sample Compartment on the Sensitivity

In this section, the impact of the radius of the sample compartment and thus the radius *r*_M_ of the medium is discussed. In [37], we have derived Equation (10) in order to improve the sensitivity of the differential transformer by an optimized distance *d*_PCB_ between the three planar PCB coils, see Figure 3. This mathematical model can be used to increase the sensitivity and the signal-to-noise ratio *SNR* by optimizing the magnetic coupling between the coils and the medium.

(10) Sκ ~ CnSnPrM,M2 LP(rP,M2+(dPCB+dM)2)32(1(rM,M2+dM2)32−1(rM,M2+(2dPCB+dM)2)32)

**Figure 7:**
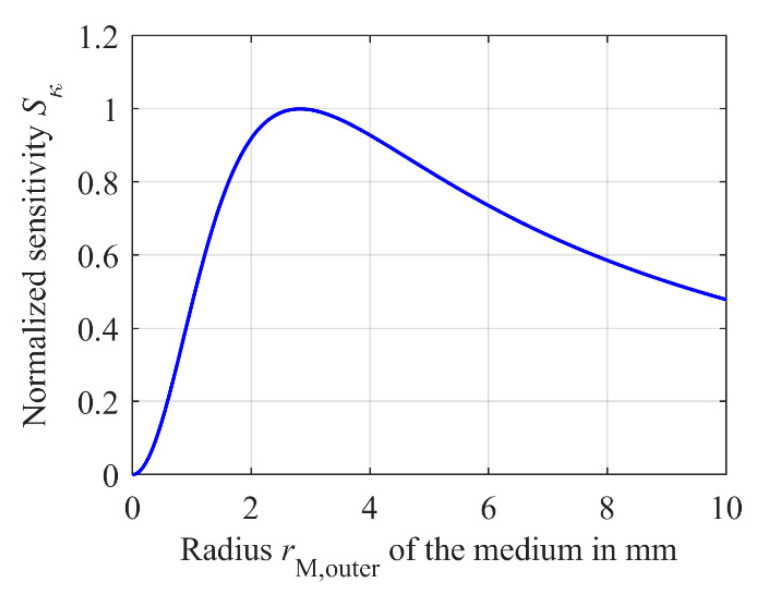
Calculated sensitivity *S*_κ_ of the differential transformer using Equation (10) normalized to the maximum sensitivity depending on the outer radius *r*_M,outer_ of the medium. If the inner radius *r*_M,inner_ of the medium is set to zero, *r*_M,outer_ = 2∙*r*_M,M_ applies. The distance *d*_PCB_ between the PCB coils is 8 mm, the distance *d*_M_ between the medium and the upper secondary coil *L*_S1_ is 1 mm.

*d*_M_ is the distance between the medium and the upper secondary coil *L*_S1_, see Figure 3. The factor *C* summarizes further constants as well as geometrical factors of the ferrite core, the angular frequency and the primary voltage, which will not be discussed here any further. Considering Equation (10), additional to the distance *d*_PCB_ the mean medium radius *r*_M,M_ is relevant. *r*_M,M_ is defined as the geometric mean value between the inner radius *r*_M,inner_ and the outer radius *r*_M,outer_ of the cylindrically shaped sample compartment, representing the boundaries of the medium. Plotting Equation (10) for *r*_M,inner_ = 0 mm versus *r*_M,outer_ and setting a distance *d*_PCB_ between the PCB coils to 8 mm and *d*_M_ to 1 mm leads to Figure 7. Setting *r*_M,inner_ to zero yields *r*_M,outer_ = 2∙*r*_M,M_.

For deriving Equation (10), the medium was considered as a coil with one winding carrying the total induced eddy and displacement currents *I*_M_ at *r*_M,M_ and thus causing a secondary field *B*_S_. Hence, *B*_S_ can be determined by using the Biot–Savart law according to Equation (6) [37] and Figure 7 and therefore Equation (10) can be interpreted accordingly. First, the amplitude of the secondary field *B*_S_ increases squared to radius *r*_M,M_. Thus, the factor *r*^2^_M,M_ in Equation (10) dominates the influence on the sensitivity *S*_κ_ for small radii, leading to an increased sensitivity increasing *r*_M,M_. However, at a certain value of *r*_M,M_, the secondary field reaches the lower secondary coil *L*_S2_, see Equation (6), resulting in a progressive penetration of *B*_S_ of both secondary coils *L*_S1_ and *L*_S2_ with increasing *r*_M,M_. Hence, a reduction of *S*_κ_ can be observed. The loss of sensitivity due progressive penetration of *B*_S_ of both secondary coils *L*_S1_ and *L*_S2_ is described in Equation (10) by the term in parentheses. However, Equation (10) only considers the geometrical effect of *r*_M,M_. The total current intensity of the eddy and displacement currents *I*_M_ was determined in [37] using Faraday’s law and the total impedance *Z*_M_ of the medium. The dependence of *Z*_M_ on the height *h*_M_ and the mean radius *r*_M,M_ of the medium was neglected. Furthermore, the complex distribution of the primary field *B*_P_ was simplified and assumed to be just oriented in positive *z*-direction within the ferrite core. These simplifications leads to a constant induced current *I*_M_ within the medium independent of the radius *r*_M,outer_. A constant current *I*_M_ independent of the radius would imply a current density *J*_M_ evenly distributed along the radius *r*_M_. The amplitude of *J*_M_ changes as a function of *r*_M,outer_. However, the current *I*_M_ is expected to grow progressively with increasing size of the medium as *Z*_M_ decreases. After a certain value of *r*_M,outer_, less current is induced in the outer area with a large radius of the medium due to the complex field distribution of *B*_P_. As a result, the current *I*_M_ should saturate.

**Figure 8:**
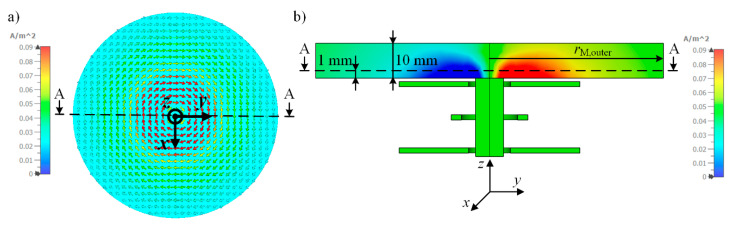
Simulation of the distribution of the current density *J*_M_ within the medium. The total height *h*_M_ is 10 mm and the outer radius *r*_M,outer_ is 50 mm. The primary coil is excited with 1 V_PP_ at 155 kHz. (**a**) The top view shows a cross section at a height of 1 mm in *z*-direction through the medium in the *x*-*y*-plane. *J*_M_ is represented by arrows. (**b**) Cross sectional view in the *y*-*z* plane. The intensity of *J*_M_ is color-coded.

Therefore, using the differential transformer model from Section 2.3, we simulate the current distribution *J*_M_ within the medium at a conductivity *κ* of the medium of 2 S/m at an excitation of the primary coil of 1 V_PP_ and a frequency of 155 kHz. Figure 8a shows a cross sectional view in the *x*-*y*-plane from the top. The simulated current density is represented by arrows and rotates around the *z*-axis. The color gradient indicates the intensity of *J*_M_. Figure 8b shows a cross sectional view in the *y*-*z*-plane. Again, the intensity *J*_M_ is given by the color gradient.

**Figure 9:**
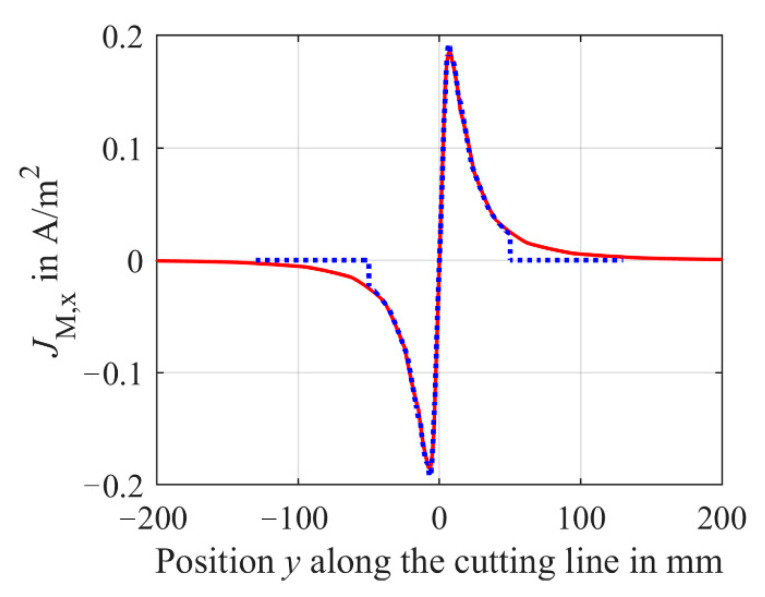
Simulated current density distribution *J*_M,x_ of the *x*-component of the induced eddy and displacement current densities along the cutting line A-A located at a height of 1 mm within the medium. For the simulations, the basic setup of the simulation model from Section 2.3 is used. The cutting line A-A is depicted in Figure 8. The total height *h*_M_ of the medium is 10 mm and the conductivity is 2 S/m. The blue dotted line shows *J*_M,x_ for an outer radius *r*_M,outer_ of the medium of 50 mm and the red solid line for *r*_M,outer_ = 200 mm. *r*_M,outer_ represents the boundaries of the sample compartment in radial direction.

Figure 9 shows the resulting current density distribution *J*_M,x_ of the *x*-component of the induced eddy and displacement current densities along the cutting line A-A located at a height of 1 mm inside the medium as depicted in Figure 8. The outer radius *r*_M,outer_ of the medium is 200 mm (Figure 9, red solid line) and 50 mm (Figure 9, blue dotted line), respectively, and the height *h*_M_ is 10 mm. These parameters represent the boundaries of the sample compartment.

Figure 9 reveals a point symmetry of the current density *J*_M,x_ to the origin, representing the center of the radially symmetric medium. Starting from the origin of the medium (x = y = 0 mm), the absolute value of the current density increases with increasing radius *r*_M_ and reaches a maximum at about 7 mm. Then, the absolute current density decreases until *J*_M,x_ approaches zero. Comparing the current densities *J*_M,x_ for sample boundaries of *r*_M,outer_ = 50 mm and *r*_M,outer_ = 200 mm, the current density *J*_M,x_ is almost independent of *r*_M,outer_ until the outer boundary *r*_M,outer_ is reached. Beyond this boundary, it is evident that the current is forced to zero. Hence, the initial assumption for Equation (10) of a evenly distributed *J*_M_ along the radius with an amplitude depending on the boundary of the sample compartment *r*_M,outer_ is not fulfilled. Thus, the total current of the induced eddy and displacement currents *I*_M_ is also not constant for different radii of the medium as long as *J*_M_ has not fully decreased to zero for very high *r*_M,outer_. Furthermore, a possible influence of different radii *r*_S,outer_ of the secondary coils *L*_S1_ and *L*_S2_ is not considered in this Equation (10).

**Figure 10:**
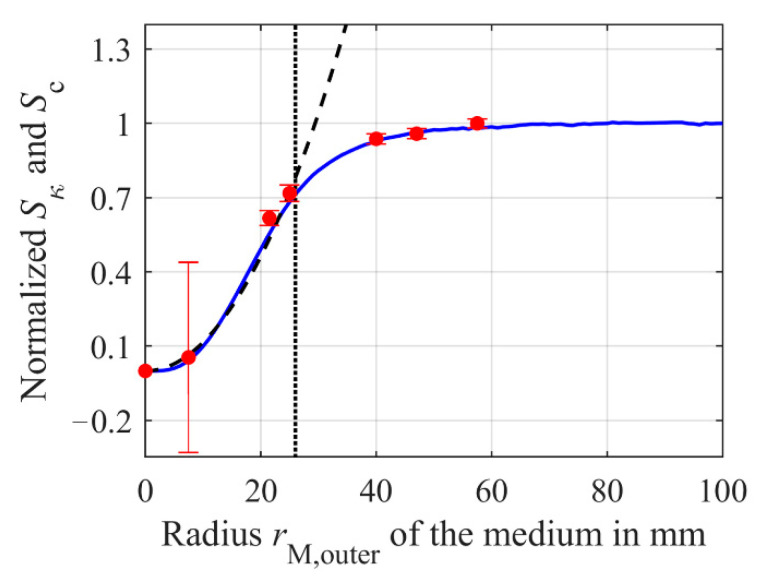
Simulated sensitivity *S*_κ_ (blue solid line) normalized to *S*_κ,max_ of 88 μV/S/m and measured sensitivity *S*_c_ (red dots) with error bars of the sensor noise translated into a concentration *c*_std_, both normalized to *S*_c,max_ of 30.98 mV/mol/L versus the outer radius *r*_M,outer_ of the medium. The basic setups from Section 2.1 and Section 2.3 were used for the measurements and simulation, respectively. To determine *S*_κ_, the conductivity of the medium in the simulation model was varied between 1 S/m and 2 S/m. *S*_c_ was determined via measurements by two different NaCl concentration within the medium (100 mmol/L and 150 mmol/L). The height *h*_M_ of the medium was 10 mm. The outer secondary coil radius *r*_S,outer_ was 26 mm in both cases and is shown as a black dotted line. The increase of *S*_κ_ and *S*_c_ up to *r*_S,outer_ can be well approximated by a quadratic function (black dashed line).

In order to determine the exact impact of the radius *r*_M,outer_ and of the outer radius *r*_S,outer_ of the secondary coils on the sensitivity, simulations are executed using the model from Section 2.3, followed by validation of the model via measurements. As for the previous simulations of the current distribution, *h*_M_ is set to 10 mm. The outer radius *r*_M,outer_ of the medium is varied from 0 mm to 100 mm. In order to determine the sensitivity *S*_κ_ according to Equation (3), each step is simulated at a conductivity of *κ* = 1 S/m and *κ* = 2 S/m. The inner radius *r*_M,inner_ is fixed to zero. The results are shown in Figure 10 as a blue solid line, normalized to the maximum sensitivity of 88 μV/S/m. As expected, the simulation shows an increasing sensitivity with increasing radius *r*_M,outer_. However, at a certain value, *S*_κ_ saturates and does not decrease.

A possible reason for the sensitivity *S*_κ_ not decreasing after a certain radius *r*_M,outer_ as shown in Figure 7 could be the more complex shape of the current density *J*_M_ within the medium. For larger radii of *r*_M,outer_, *J*_M_ decreases towards zero after its maximum at about 7 mm and thus contributes less to the secondary field *B*_S_. In addition, due to the inhomogeneous distribution of *J*_M_, the mean radius *r*_M,M_ of the medium cannot be calculated simply from the geometric mean value of *r*_M,inner_ and *r*_M,outer_. As *J*_M_ decreases for larger *r*_M,outer_, *r*_M,M_ increases much slower than expected, causing less penetration of *B*_S_ into the lower secondary coil *L*_S2_, as *B*_S_ does not extend that far along the *z*-direction, see Equation (6). This results in a saturation of *S*_κ_ instead of a reduction for larger *r*_M,outer_. For small *r*_M,outer_, the simulated characteristic of the sensitivity of the differential transformer corresponds in good approximation to the expected behavior shown in Figure 7 and can therefore be approximated well by a quadratic function *f*(*r*_M,outer_) = *a*∙*r*^2^_M,outer_ (Figure 10, black dashed line). Fitting in the range of *r*_M,outer_ = 0 mm to *r*_M,outer_ = 26 mm leads to *a* = 102.4 × 10^−6^ mVS/(mol∙mm^2^) having a coefficient of determination R^2^ of 0.9925. For *r*_M,outer_ > 26 mm, *f*(*r*_M,outer_) increasingly deviates from *S*_κ_. This value of *r*_M,outer_ is indicated by the black dotted line in Figure 10 and represents the outer radius *r*_s,outer_ of the secondary coils *L*_S1_ and *L*_S2_. The impact of the secondary coil radius *r*_S,outer_ will be investigated in more detail later. First, the simulation results have to be validated by measurements.

Therefore, similar to Section 3.1.1, the differential transformer described in Section 2.1 is driven with a voltage *U*_P_ of 1 V_PP_ at a frequency *f* of 155 kHz. The medium is placed above the differential transformer and is located in sample compartments with fixed height *h*_M_ of 10 mm and variable diameters of 15 mm, 43 mm, 50 mm, 80 mm, 94 mm, and 115 mm. The sample solutions inside the compartments contain NaCl with concentrations of *c*_1_ = 100 mmol/L and *c*_2_ = 150 mmol/L, enabling the calculation of sensitivity *S*_c_ for each radius. The measured results are normalized to the maximum sensitivity of *S*_c_ = 31 mV/mol/L and are shown in Figure 10 as red dots. As can be seen, the results correspond well with the simulations, meaning the simulation model can be considered as validated. Similar to the simulations, the sensitivity initially increases with increasing radius of the medium. As in Section 3.1.1, we also investigate whether the precision can be enhanced by the increased sensitivity. Therefore, the noise of the output voltage was determined by the standard deviations of *Im*{*U*_S_} at different radii *r*_M,outer_. The results can be found in Table 2. Table 2 reveals no correlation between the noise and *r*_M,outer_. The standard deviation of the concentration *c*_std_ was calculated by dividing the standard deviation of *Im*{*U*_S_} by the respective sensitivity *S*_c_. Due to the initial low sensitivity at *r*_M_ = 7.5 mm, the standard deviation of concentration *c*_std_ is 11.9 mmol/L. Comparing this to a radius of 57 mm, *c*_std_ is only 0.57 mmol/L, which is a significant improvement in the precision of the differential transformer. The relative error bars normalized to *S*_c,max_ are also illustrated in Figure 10.

**Figure 11:**
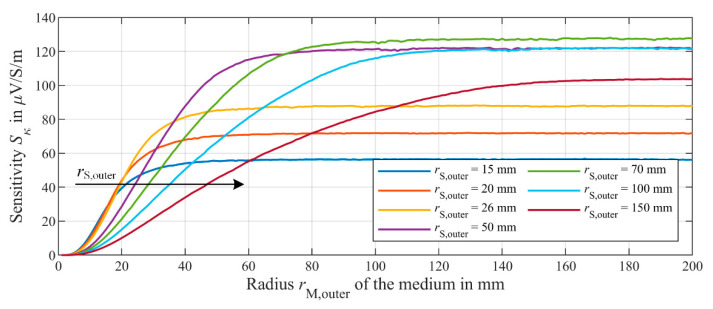
Dependence of the simulated sensitivity *S*_κ_ on the outer radius *r*_M,outer_ of the medium at different outer secondary coil radii *r*_S,outer_ of the secondary coils *L*_S1_ and *L*_S2_ as parameter. The height *h*_M_ of the medium is 10 mm. While changing *r*_S,outer_, the number of windings of the secondary coils *L*_S1_ and *L*_S2_ are constant at 542 each.

As already mentioned, the increase of both the simulated sensitivity *S*_κ_ as well as the measured sensitivity *S*_c_ saturate at an outer radius of the medium *r*_M,outer_ of about 26 mm. This corresponds exactly to the outer radius *r*_S,outer_ of the secondary coils *L*_S1_ and *L*_S2_. Therefore, the validated simulation model is used to investigate how *r*_S,outer_ affects *S*_κ_ and if there is a correlation between *r*_S,outer_ and the saturation radius. In this simulation, *r*_S,outer_ is used as parameter between 15 mm and 150 mm. Thereby, only the radius *r*_S,outer_ of the secondary coils are changed. The number of windings *n*_S_ of *L*_S1_ and *L*_S2_ are kept constant at 542. This means, for example, that the windings at *r*_S,outer_ = 15 mm spread over a much smaller area than at *r*_S,outer_ = 150 mm. The results of this simulation are shown in Figure 11.

As depicted in Figure 11, the initial increase of *S*_κ_ can approximated by a quadratic function *f*(*r*_M,outer_) = *a*∙*r*^2^_M,outer_ for all secondary coil radii *r*_S,outer_. However, for very large *r*_S,outer_ of—e.g., 150 mm—a reasonable fit can only be realized up to *r*_M,outer_ of about 40 mm, since the simulated *S*_κ_ increasingly deviates from *f*(*r*_M,outer_) for larger *r*_M,outer_. This is probably due to the increasing decline of *J*_M_ at larger radii. Furthermore, the simulations indicate a decrease of the initial slop of *S*_κ_, i.e., the factor *a* of the quadratic function *f*(*r*_M,outer_) becomes smaller, for larger secondary coil radii *r*_S,outer_. Hence, the sensitivity curve is shifted towards larger medium radii *r*_M,outer_ indicated by the arrow in Figure 11. A possible reason for this could be the reduction of the number of windings *n*_S_ of the secondary coils effectively involved in the magnetic coupling with the secondary field *B*_S_. Since the divergence of magnetic fields must always be zero, the field lines are closed. The secondary field *B*_S_ propagates from the medium inside the ferrite core in negative *z*-direction, towards the secondary coils. At larger radial distance to the ferrite core, the field lines turn back in positive *z*-direction. The outer windings of the secondary coils with large *r*_S,outer_ are thus penetrated by both the outgoing and returning field and causing a reduced net flux, so that no voltage is induced. These outer windings are effectively not involved in the coupling with the secondary field. By replacing the secondary inductance *L*_S_ with the proportionality *L*_S_ ~ *n*_S_^2^ in Equation (10), Equation (11) is obtained. Equation (11) indicates a dependency of the winding ratio between the primary coil *n*_P_ and the secondary coil *n*_S_. Comparable to an ordinary transformer, lowering the effective *n*_S_ cause a reduction in the sensitivity.

(11) $$S_{\kappa }\;\sim \;\frac{n_{S}}{n_{P}}$$

**Figure 12:**
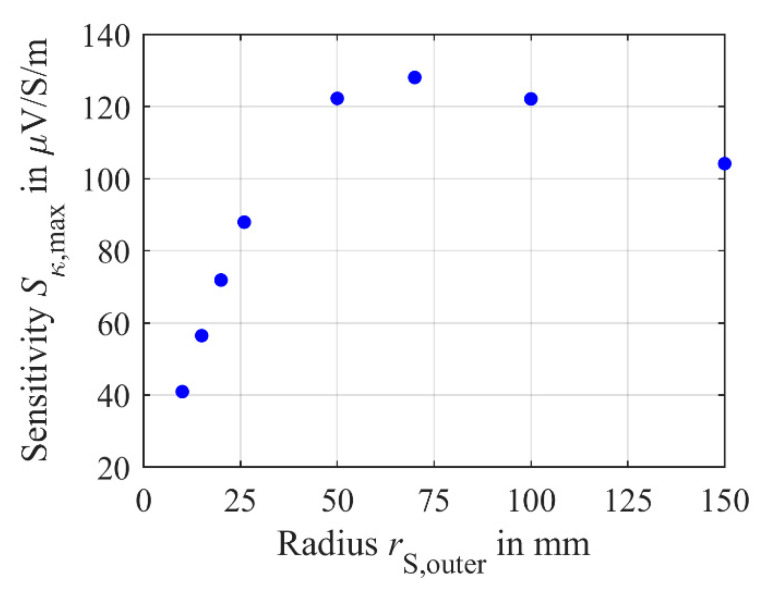
Maximum simulated sensitivity *S*_κ,max_ versus the radius *r*_S,outer_ of the secondary coils *L*_S1_ and *L*_S2_.

Nevertheless, the respective outer secondary coil radius *r*_S,outer_ always corresponds very well with saturation radius regarding the sensitivity *S*_κ_, meaning that there is a direct correlation between *r*_S,outer_ and the saturation. A possible reason for this could be the reduced contribution of the circular currents with very large radius *r*_M_ to the secondary magnetic field *B*_S_ at the origin of the medium at x = y = 0 mm propagating over the ferrite core in negative *z*-direction. Equation (6) supports this assumption, since the contribution of the circular currents to *B*_S_ at the origin decreases with *r*_M_^−1^. However, leakage fields occur in the immediate surroundings of currents circulating at the outer boundary of the medium, although with decreasing amplitude due to the declining current density *J*_M_. These leakage fields predominantly close directly around the current path and do not noticeably penetrate the ferrite core. Due to the small distance between the medium and the secondary coil *L*_S1_, only *L*_S1_ is penetrated by these leakage fields. If the outer radius *r*_M,outer_ of the medium exceeds the outer radius *r*_S,outer_ of the secondary coil, such additional currents with larger radius *r*_M_ than *r*_S,outer_ couple increasingly less with *L*_S1_, leading to the saturation of the sensitivity. Therefore, an increase of *r*_S,outer_ leads to more leakage fields being collected by *L*_S1_ and thus to an increased maximum sensitivity *S*_κ,max_. This increase in maximum sensitivity *S*_κ,max_ can be observed up to an *r*_S,outer_ of 70 mm. However, for higher *r*_S,outer_ *S*_κ,max_ starts to decreases again as shown in Figure 12. Here, the maximum simulated sensitivity *S*_κ,max_ is plotted versus the outer radius *r*_S,outer_ of the secondary coils *L*_S1_ and *L*_S2_.

Again, this decrease in the maximum sensitivity *S*_κ,max_ for higher secondary radii *r*_S,outer_ can be described by the reduction in the effective number of windings *n*_S_ involved in the coupling. At large radii, the current within the medium has nearly approached zero. Therefore, an increase of *r*_M,outer_ has only a negligible effect, since almost no current exists. If the outer secondary coil radius *r*_S,outer_ exceeds the radius in the medium where almost no current flows, the effective number of windings *n*_S_ is irrevocably reduced due to the same effect described before. Thus, to obtain an optimum design of the differential transformer in terms of sensitivity, the secondary coil radius *r*_S,outer_ and radius of the medium must be carefully adjusted to each other. For example, a restricted radius *r*_M,outer_ of the sample due to a limited sample volume needs a secondary coil radius close to *r*_M,outer_. If *r*_M,outer_ is not limited by the application, the secondary coil radius *r*_S,outer_ have to be carefully adjusted to achieve high maximum sensitivity *S*_κ,max_.

In the section above, the dependence of the sensitivities *S*_κ_ and *S*_c_ of the differential transformer on the radius of the medium has been analyzed. A good approximation of the initial increase of the sensitivities *S*_κ_ and *S*_c_ by increasing *r*_M,outer_ with a quadratic function was found. Thus, an increase of *r*_M,outer_ has significant impact on the sensitivity. For a constant number of windings of the secondary coils, the slope of this approximating function depends on the radius *r*_S,outer_ of the secondary coil. Increasing *r*_S,outer_ to *r*_S,outer_ ≥ *r*_M,outer_ results in the outer windings of the secondary coil not participating in the magnetic coupling between the medium and the secondary coil. Hence, the effective number of windings *n*_S_ of the secondary coil decreases, causing a decrease in sensitivity. The same effect of uncoupled windings *n*_S_ also occurs when *r*_S,outer_ is increased over the radius where only negligible eddy and displacement currents circulate even for *r*_S,outer_ < *r*_M,outer_. If the radius *r*_M,outer_ of the medium exceeds the radius of the secondary coil, the sensitivity saturates, i.e., a further increase of *r*_M,outer_ has no effect on *S*_κ_ and *S*_c_. Thus, if *r*_M,outer_ exceeds *r*_S,outer_, the maximum sensitivity is reached. In experimental investigations, an increase of *r*_M,outer_ seems useful, since the sensitivity significantly increases, but no correlation of the noise to *r*_M,outer_ is observed. Thus, increasing the sensitivity by increasing *r*_M,outer_ is a reasonable solution to improve the signal-to-noise ratio *SNR*.

## 4. Conclusions

In this work, we have investigated the influence of the sample compartment geometry on the sensitivities *S*_κ_ and *S*_c_ and the precision of a differential transformer. Therefore, we have addressed various design parameters with the use of the simulation software CST-EM Studios. The experimental investigations were conducted by using a PCB differential transformer.

First, the depth of field penetration was considered. The findings have revealed a variety of applications, where the standard depth of penetration *δ*_S_ or skin depth cannot be used to calculate the depth of penetration of the differential transformer as it is usually used for other eddy current sensors, e.g., for non-destructive material testing. Examples of these applications are continuous non-invasive monitoring of sodium concentration in blood, quality monitoring of liquids and monitoring of processes in bioreactors. Since the conductivity of the medium is relatively low here, only negligible attenuation occurs within the medium. Instead, the field characteristic of the exited primary coil is much more important. Thus, we have establish a new equation using the Biot–Savart law, allowing us to calculate the true depth of penetration *δ*_T_ of the differential transformer as a function of the mean primary coil radius *r*_P,M_. This equation can be used as long as the ratio of *r*_P,M_ to the standard depth of penetration *δ*_S_ is lower than 10^−1^. The true depth of penetration *δ*_T_ calculated for the used PCB differential transformer is 7 mm. The depth of penetration determined by simulations and measurements is 7.4 mm. Thus, the simulated and measured *δ*_T_ is in good agreement with the calculated *δ*_T_. Furthermore, the results show an increasing sensitivities *S*_κ_ and *S*_c_ by increasing the height *h*_M_ of the medium and thus the sample compartment up to *h*_M_ = 3∙*δ*_T_, while the noise is not affected by *h*_M_. Therefore, the signal-to-noise ratio *SNR* improves by increasing *h*_M_. For example, converting the noise of the output signal of the differential transformer to a concentration, the standard deviation of the concentration can be reduced from 0.81 mmol/L at *h*_M_ = 5 mm to 0.44 mmol/L at *h*_M_ = 50 mm and thus improving the precision.

Secondly, the impact of the outer radius *r*_M,outer_ of the medium and thus sample compartment was investigated. The results show an initial quadratic increase of the sensitivities *S*_κ_ and *S*_c_ with the radius *r*_M,outer_. If *r*_M,outer_ exceeds the outer radius *r*_S,outer_ of the secondary coils, the sensitivities saturates, as from this point no relevant coupling exists between the additional secondary field *B*_S_ and the secondary coils. In general, the results reveal a complex interaction between the radius of the medium and the radius of the secondary coils. For instance, if the outer radius *r*_S,outer_ of the secondary coil is larger than *r*_M,outer_, or if the radius of the secondary coil is so large that no significant eddy and displacement currents are induced within the medium, the outer secondary coil windings are no longer involved in relevant magnetic coupling between the coil and the medium. Thus, the effective windings *n*_S_ of the secondary coils is reduced and consequently the sensitivity. As with the depth of penetration, increasing the sensitivity by increasing the radius of the medium and thus sample compartment the signal-to-noise ratio improves, since no correlation between the standard deviation of the noise and the radius of the medium was observed. For example, considering an outer radius of the medium of 7.5 mm, the standard deviation of the noise converted into a concentration is 11.9 mmol/L. In contrast, an outer radius of the medium of 57 mm results in a standard deviation of the noise converted into a concentration of only 0.57 mmol/L. Thus, by increasing the outer radius of the medium, the precision of the differential transformer can be significantly improved.

In future work, we will investigate whether a sample can also be characterized directly through a hose system (patent pending). In particular, the winding technique of the hose around an extended ferrite core could affect the sensitivity. The advantage of this approach would be in-line measurement of hose-guided samples without the need to leave the hose system and to be passed into a flow chamber.

## Figures and Tables

**Table 1:** Measured standard deviation of the output voltage *Im*{*U_S_*} of the differential transformer, corresponding sensitivity *S*_c_ and the resulting standard deviation of the measured concentration *c*_std_ depending on the height *h*_M_ of the medium. The experimental measured data are included in Figure 6 (red).

*h*_M_ in mm	Standard Deviation of *Im*{*U_S_*} in μV	*S*_c_ in mV/mol/L	Standard Deviation of *c*_std_ in mmol/L
0	18.82	0	-
5	18.07	22.28	0.81
10	18.43	29.69	0.62
15	19.65	32.82	0.60
20	19.02	35.00	0.54
25	17.26	36.50	0.47
30	20.36	36.34	0.56
35	19.52	36.10	0.54
40	18.88	37.50	0.50
50	17.10	38.74	0.44

**Table 2:** Measured standard deviation of the output voltage *Im*{*U_S_*} of the differential transformer, corresponding sensitivity *S*_c_ and the resulting standard deviation of the measured concentration *c*_std_ depending on the outer radius *r*_M,outer_ of the medium. The experimental measured data are included in Figure 10 (red).

*r*_M,outer_ in mm	Standard Deviation of *Im*{*U_S_*} in μV	*S*_c_ in mV/mol/L	Standard Deviation of *c*_std_ in mmol/L
0	18.82	0	-
7.5	20.23	1.7	11.9
25	23.31	22.24	1.05
40	18.43	29.04	0.63
47	18.43	29.69	0.62
57	17.67	30.98	0.57

## Data Availability

The data presented in this study are available on request from the corresponding author.
